# Monthly Report of Diseases

**Published:** 1801-03

**Authors:** 


### MONTHLY REPORT of DISEASES,

Admitted under the Care of the Physicians of the Finsbury Dispensary, St. John's Square, Clerkenwell.
From Jan. 20, to Feb. 20, 1801.

| | No. of Cafes. | | No. of Cafes. |
|---|---|---|---|
| Hypochondriafis and Dyfpepfia | 36 | Continued Fever | 46 |
| Afthenia | 59 | Eryfipelas | 7 |
| Hyfteria | 2 | Cynanche Tonfillarum | 2 |
| Epilepfy | 1 | Pneumonia | 1 |
| Vertigo | 1 | Phthifis Pulmonalis | 4 |
| Cephalæa | 3 | Cough and Dyfpnœa | 23 |
| Anafarca | 5 | Diarrhœa | 14 |
| Infantile Difeafes | 23 | Chlorofis and Amenorrhœa | 19 |
| Chronic Eruptions | 19 | Menorrhagia | 10 |

It is generally fuppofed that what are called nervous affections are almoft exclufively confined to the fuperior orders of fociety: fo so far, however, from being the exclusive property of the rich and the luxurious, they appear, in some shape or other, to prevail in an equal proportion among the humblest classes of the community. The nerves of the poor are subject to the same morbid vibrations, and their imaginations to as great a variety of ridiculous and tormenting caprices, as even those are liable to that move in the very highest circles of the fashionable world. Cases of this description, so great a number of which have come under observation during the last year, have been remarkable for the multiplicity and diversity of their symptoms. Some have apprehended the near approach of death, when, to an impartial observer, they shewed every symptom that could indicate health, or that could give a promise of longevity. Some were continually haunted by frightful spectres; some fancied that there was something alive within them*; others, that they had no inside; as well as a great number of corporeal deficiencies and complaints which were entirely absent, and the presence of which there was not the slightest reason for suspecting.

No man has greater opportunities of observing the connection between the prevailing diseases and the various states of the weather, than the physician whose humanity or professional duty calls him to the relief of those classes of society which are most exposed to its influence. It is the peculiar privilege of Dispensary-practice that, being conducted upon a scale of vast extent, it presents an immense multitude of facts, from which this connection may be easily and satisfactorily traced. To note the effects of climate on the human frame, an enquiry of no less importance in a moral than in a physical point of view, is therefore the especial province of the practitioner, to whom this ample field of observation lies open. The general conclusion that will be found to result from the enquiry is, that no state of weather is equally salutary to every variety of constitution, or conducive to the relief of every species of complaint. A mild winter, by removing many causes of illness to which the poor are particularly exposed, is extensively beneficial; while, on the other hand, it is injurious almost to an equal extent, by impairing the vigour of the frame, and thus predisposing it to the long train of disease to which debility is the source.

The extraordinary warmth of the present winter, which has in some measure disturbed the natural order of the seasons, has occasioned a corresponding deviation in the usual course and succession of diseases. As the protracted autumn had prolonged the disposition to contagious fever, so the premature revival of spring has diminished the frequency and softened the severity of pulmonary complaints.

The late frost has scarcely been of sufficient continuance to arrest the

* In one or two female cases, indeed, it turned out that *this* fancy was not altogether without foundation.

the progrefs of febrile infection. Seldom, indeed, does the fudden occurrence of great cold, after a fever has once taken full poffeffion of the conftitution, immediately tend to mitigate the violence of its fymptoms. This fact may, perhaps, admit of explanation, when we confider the rooted prejudice of the lower claffes in favour of accumulating warmth around a fick bed. Cold weather being always more feverely felt on its fudden arrival, than when a gradual approach has prepared for the encounter, will tend, in the former cafe, to infpire additional anxiety to obtain effectual protection againft its attacks. The wretched patient, wafting under a burning fever, will often be overwhelmed, by the too officious care of his relations, with a fuperfluous load of bed-cloaths, and defended, with ill-judged zeal, from the falutary renewal of air. Heat, thus artificially excited, expends in fruitlefs wafte the laft remains of vitality; and an atmofphere, thus ftagnant and replete with poifon, more fatally malignant than the difeafe, extinguifhes in filence the dimly glimmering flame of life.

Pulmonary complaints have been more frequent fince the late alteration in the weather. Few difeafes require more fagacity in detecting their nature, or greater accuracy in difcriminating their varieties, than thofe that affect the organs of refpiration: in none is it of greater importance that the diagnofis fhould be juft. No miftake is more likely to be attended with fuch fatal confequence to the life of the patient than an error of judgment with regard to this particular. The fame remedy, which will in one cafe fave, would, if applied in another, inevitably deftroy. Great attention is requifite to recognife the flow and infidious approach of peripneumonia notha; a difeafe, which, in this city, fo often fupervenes upon an ordinary catarrh. Hackney-coachmen are peculiarly liable to its attacks. Expofed to all the viciffitudes of an inconftant climate, with little general exercife of body, and with none that tends to preferve the feet in a due degree of warmth, it frequently in them affumes the leading characters of the true pleurify. A phyfician, who was to have recourfe to the lancet, would learn too late, by the aggravation of every fymptom, and the fpeedy death of his patient, the fatal and irretrievable error he had committed. Bleeding is a remedy feldom applicable to the difeafes which afflict the poor of the metropolis. Their general character has been for a long time paft complicated with fymptoms of debility. Of late, indeed, many caufes have confpired with the warmth of the feafon, to enervate the once robuft habits of our countrymen. Thofe circumftances which produced fuch ravages in former years, have, it is true, operated with inferior force. But little has it availed the poor that they have experienced lefs inclemency from the elements, while at the fame time they have wanted internal fupport, as well as exterior protection againft the viciffitudes of our atmofphere. They have had to ftruggle with an unprecedented degree of hunger, anxiety, and fatigue. Under the accumulated preffure of hardfhips like thefe, is it to be wondered at that difeafe has fpread fo widely, and yielded fo many victims to the grafp of death? Is it to be wondered at that the mournful mournful catalogue of infirmities, which each fucceeding period obtrudes upon our view, fhould in every feafon, and in every country, ftill prefent the fame picture of calamity, ftill rehearfe the fame endlefs tale of human mifery?

*Red Lyon Square.* J. R.

